# Auxin Is Involved in Magnesium-Mediated Photoprotection in Photosystems of Alfalfa Seedlings Under Aluminum Stress

**DOI:** 10.3389/fpls.2020.00746

**Published:** 2020-06-03

**Authors:** Liantai Su, Aimin Lv, Wuwu Wen, Peng Zhou, Yuan An

**Affiliations:** ^1^School of Agriculture and Biology, Shanghai Jiao Tong University, Shanghai, China; ^2^Key Laboratory of Urban Agriculture, Ministry of Agriculture, Shanghai, China

**Keywords:** aluminum, cyclic electron flow, IAA, magnesium, proton motive force, proton gradient

## Abstract

The objective of this study was to investigate the effects of Mg and IAA on the photosystems of Al-stressed alfalfa (*Medicago sativa* L.). Alfalfa seedlings with or without apical buds were exposed to solutions fully mixed with 0 or 100 μM AlCl_3_ and 0 or 50 μM MgCl_2_ followed by foliar spray with water or IAA. Results from seedlings with apical buds showed that application of Mg and IAA either alone or combine greatly alleviated the Al-induced damage on photosystems. The values of photosynthetic rate (Pn), effective quantum yields [Y(I) and Y(II)] and electron transfer rates (ETRI and ETRII), proton motive force (*pmf*), cyclic electron flow (CEF), proton efflux rate (g_H_^+^), and activities of ATP synthase and PM H^+^-ATPase significantly increased, and proton gradient (ΔpH*_pmf_*) between lumen and stroma decreased under Al stress. After removing apical buds of seedlings, the Y(I), Y(II), ETRI, ETRII, *pmf*, and g_H_^+^ under exogenous spraying IAA significantly increased, and ΔpH*_pmf_* significantly decreased in Mg addition than Al treatment alone, but they were no significant difference under none spraying IAA. The interaction of Mg and IAA directly increased quantum yields and electron transfer rates, and decreased O_2_^–^ accumulation in Al-stressed seedlings with or without apical buds. These results suggest that IAA involves in Mg alleviation of Al-induced photosystem damage via increasing *pmf* and PM H^+^-ATPase activity, and decreasing ΔpH*_pmf_*.

## Introduction

Aluminum (Al) is the most abundant metal and is widely distributed in nature in the form of silicates or other deposits. Excessive soluble Al^3+^ content in acidic soils is highly phytotoxic for crop growth and causes a number of adverse effects on physiological and biochemical processes. A primary symptom of Al toxicity in plants is the reduction of root growth, followed by limiting nutrient uptake (Kochian et al., 2015; Julietta et al., 2016; Fan et al., 2019). Moreover, excess Al induced reduction in CO_2_ assimilation has been found in many crops like rye (Silva et al., 2012) and wheat (Julietta et al., 2016). The main photosynthetic apparatus consists of the light-harvesting PSI, PSII, and cytochrome b_6_f (Mikko and Aro, 2014). PSII, the “engine of life,” is the photosynthetic enzyme that uses sunlight energy to extract electrons from water for conversion of inorganic molecules into organic molecules (James, 2009). PSII has three functional domains: (i) the antenna of chlorophyll (Chl) and other pigments which absorb and transfer photon energy to (ii) the reaction center (RC) where the excited state electron from a special pair of Chl a molecules (P680) is transferred to a series of electron acceptors, and (iii) the oxygen evolving complex (OEC) where the electrons are extracted from water (Allen et al., 2015). PSI is a multi-subunit pigment–protein complex embedded in the thylakoid membrane of the chloroplast. It catalyzes light-driven electron transfer from PC [a luminal mobile protein that receives electrons from cytochrome b_6_/f (Cyt b_6_/f)] to Fd located at the stromal side. The RC of PSI is P700, which is an electron donor that consists of a dimer of Chl a molecule (Hasni et al., 2015a).

The electron transport from PSII to PSI is tightly coupled with the generation of thylakoid *pmf*, composed of both electric field (ΔΨ) and pH (ΔpH) gradients between lumen and stroma (Huang et al., 2017). Under normal conditions, pH is 7.3–7.6 in the cytoplasm, 4.5–5.9 in vacuoles, ∼7 in mitochondria, 7.2–7.8 in chloroplasts, 5.8–6.8 in lumen, ∼7 in stroma, and ∼5.5 in apoplasm (Thomas and Anja, 2014; Wang P. et al., 2016). These differences of pH values among organelles form a relatively stabilized H^+^ environment for cells to fulfill a series of physiological and chemistry progresses. In the thylakoid, a low level of lumen acidification regulates the oxidation of plastoquinol in the b_6_f, which reduces electron transfer from PSII to PSI and prevents electron accumulation in PSI to photo-protect PSI. The *pmf* and its partitioning into ΔΨ and ΔpH components are regulated to maintain the lumen pH above 5.8 in the normal, in which it can regulate photoprotection for PSI (Geoffry et al., 2016). Many studies have shown that excess Al inhibits photosynthetic electron transport in PSII and PSI, closes their RCs, and reduces the amount of active population of P700 (Hasni et al., 2015b; Julietta et al., 2016), thereby decreases photosynthesis.

It has been estimated that, as a key constituent of Chl molecules, up to 35% of total Mg content in plants is in chloroplasts (Cakmak and Yazici, 2010; Nèjia et al., 2015). Thus, Mg is critical for plant photosynthesis (Jin et al., 2016), formation of reactive oxygen species (Cakmak and Kirkby, 2008), and protein biosynthesis and enzyme activation (Peng et al., 2015). Even so, little attention has been paid to Mg in the last decades compared with other nutrient elements. Therefore, Mg is recently considered to be “the forgotten element” (Cakmak and Yazici, 2010).

Plants often suffer from Mg deficiency in acidic or sandy soils, because soluble Mg easily leaches from these soils and its absorption is intensively antagonized by the absorption of other cations such as aluminum, ammonium, and potassium (Yang et al., 2007). Chl content is one of the most sensitive responses to Mg deficiency. Decreased Chl content and enhanced Chl a/b ratio were observed under Mg deficiency in plants (Laing et al., 2000). Mg deficiency also adversely affects the activity of ribulose-1,5-bisphosphate carboxylase, decreases the Fv/Fm ratio and relative amount of oxidizable P700, and impairs the linear photosynthetic electron transport rate (ETR), as a result, leads to reduce CO_2_ assimilation (Tang et al., 2012). These negative effects often associated with loss of PSII antenna or changes in photosystem stoichiometry in favor of PSI due to different sensitivity of PSI and PSII to Mg deficiency (Tang et al., 2012; Nèjia et al., 2015; Jin et al., 2016).

Magnesium-mediated alleviation of aluminum toxicity has been observed in a number of plant species. Al-resistant genotypes of *Arabidopsis* maintain higher Mg accumulation and have higher Mg influx and intracellular Mg concentration in comparison to Al-sensitive genotypes. Increased Mg uptake correlates with an enhanced capacity of *Arabidopsis* to cope with Al stress (Bose et al., 2013). MGT1 is a transporter for Mg uptake in plants, and overexpression of *OsMGT1* and *AtMGT1* genes in plants is required for conferring Al tolerance in rice and *Arabidopsis* (Deng et al., 2006; Chang et al., 2012). Bose et al. (2011) suggested that mechanisms underlying the alleviating effects of Mg on Al toxicity might include (i) increased synthesis and exudation of organic acid anions; (ii) enhanced acid phosphatase activity; (iii) maintenance of H^+^-ATPase activity and cytoplasmic pH regulation; and (iv) protection against reactive oxygen species. Mg can alleviate Al rhizotoxicity by increasing PM H^+^-ATPase activity and citrate exudation in *Vicia faba* L. and rice bean roots (Yang et al., 2007; Chen et al., 2015). Although numerous studies have reported the detailed information about the target and action mode of Mg on alleviation of Al-induced damage in plants, its effective mechanism in photosynthesis remains unclear.

Auxin plays a central role in plant adaptation to environmental stress by mediating photosynthesis, development, and nutrient allocation (Peleg and Blumwald, 2011). For example, exogenous application of indole-3-acetic acid (IAA) enhances the Pn, stomatal conductance and transpiration rate of *Panax ginseng* (Li and Xu, 2014). IAA increases the Pn of Cd-treated eggplant seedlings, which is related to restore functional and structural attributes of PSII such as quantum yield, size and number of photosynthetic center, and water splitting complex (Shikha and Sheo, 2015). Expression levels of many genes responded to iron deficiency are upregulated in shoots under exogenous application of auxin, and overexpression of these genes alters the performance of photosynthetic parameters under Fe deficiency (Liu et al., 2015). Thus, there is a crosstalk between auxin signaling and photosynthetic depression induced by metal cation deficiency. However, how auxin signaling is involved in Al-induced inhibition in photosystems is unknown.

Our previous study showed that exogenous application of IAA increased H^+^-ATPase activity in Al-stressed alfalfa roots, and promoted H^+^ secretion from root tips (Wang et al., 2017). The IAA-activated H^+^-ATPase activity involved in Mg uptake and distribution, accompanied with citrate exudation in Al stressed *V. faba* roots (Takahashi et al., 2012). Because both of Mg and IAA have the function of regulating H^+^-ATPase activity and cytoplasmic pH, we speculated that there was a crosstalk between Mg and IAA on affecting photosynthetic apparatus of alfalfa to cope with Al stress. Thus, we focused our study on: (1) the effects of Mg and IAA on photosynthesis and photosystems of alfalfa under Al stress and (2) the interaction between Mg and IAA on photosynthesis and photosystems of alfalfa under Al stress.

## Materials and Methods

### Plant Materials and Growth Conditions

The alfalfa (*Medicago sativa* L.) variety, WL525, was used as plant material in present study. Seeds germinated on a filter paper moistened regularly with ½-strength Hoagland’s nutrient solution at 25°C. The uniform seedlings were transplanted to a foam board (12 holes/plate; six seedlings/hole) floating on aerated ½-strength Hoagland’s nutrient solution (pH 5.8) in plastic containers. The solutions were replaced every 2 days. All seedlings were grown in a greenhouse for 4 days with a temperature regime of 25/20°C (day/night), 14-h photoperiod, and a photon flux density of 400 μmol m^–2^ s^–1^, and then seedlings were treated by Mg and IAA as following experimental design.

### Treatments and Experimental Design

A simple solution [1.5 mM Ca(NO_3_)_2_] was used in the experiment rather than used ½-strength Hoagland’s nutrient solution in order to avoid cations in Hoagland’s nutrient to inactivate with Mg (Ishikawa and Wagatsuma, 1998). The dosage of 50 μM Mg was used in the follow-up experiments according to our preliminary experiments (Supplementary Figure S1).

#### Experimental One

Effects of Mg^2+^ and IAA on photosystems under Al stress. The above seedlings were treated with 1.5 mM Ca(NO_3_)_2_ solution (pH 4.5) supplemented with the combination of 0 or 100 μM AlCl_3_ and 0 or 50 μM MgCl_2_, and then foliar sprayed with 2 mL water (pH 6.0) or 2 mL of 6 mg L^–1^ IAA (Sigma, St. Louis, MO, United States) every 2 days. This led to five treatments in total: control (pH4.5), AlCl_3_ with or without spraying IAA (pH4.5+Al, pH4.5+Al+IAA) and AlCl_3_ and MgCl_2_ with or without spraying IAA (pH4.5+Al+Mg, pH4.5+Al+Mg+IAA). The fresh weights of roots and shoots were measured at the third and sixth days after the initiation of treatments, and plant samples were collected for further analysis.

#### Experimental Two

Our previous study showed that IAA was synthetized in apical bud of alfalfa, and removing buds greatly decrease the IAA content in root tips (Wang S. Y. et al., 2016). Thus, an experiment of removing apical buds of seedlings was conducted to further explore the interaction of Mg^2+^ and IAA on protecting photosystems under Al stress. Apical buds of seedlings were removed, and then the seedlings were divided into two groups after 2 days. One group was sprayed with water (-IAA), and the other group was sprayed with IAA (+IAA, 6 mg L^–1^). The Al and Mg addition was the same as experimental one. Six treatments were in total: seedlings treated with or without spraying IAA (pH4.5-IAA, pH4.5+IAA), 100 μM AlCl_3_ with or without spraying IAA (pH4.5+Al-IAA, pH4.5+Al+IAA) and 100 μM AlCl_3_ and 50 μM MgCl_2_ with or without spraying IAA (pH4.5+Al+Mg-IAA, pH4.5+Al+Mg+IAA). After 3 days, the parameters of PSI and PSII, and contents of NADP^+^, NADPH, and O_2_^–^ were measured.

### Chlorophyll Content Measurement

Fresh leaves were sampled from each treatment, and their Chls were extracted with 80% acetone at room temperature in dark for 24 h. Chl content was determined photometrically by measuring absorption at 663 and 645 nm using a microplate reader (Synergy2, BioTek, United States), and was then calculated using the following formulae.

Chlorophyll a (mg/g FW) = (12.7 × A663− 2.69 × A645) × V/1000/FW (g)

Chlorophyll b (mg/g FW) = (22.9 × A645−4.68 × A663) × V/1000/FW (g)

Total Chl (mg/g FW) = (20.21 × A645+8.02 × A663) × V/1000/FW (g)

Where V refers to the volume (mL) of extracting solution.

### Measurement of Net Photosynthetic Rate, Chlorophyll Fluorescence, and P700 Parameters

Five leaves per treatment were used for measuring P_n_ with a Portable Gas Exchange Fluorescence Systems GFS-3000 (Walz, Effeltrich, Germany) under the light intensity at 800 μmol m^–2^ s^–1^ and CO_2_ concentration of 400 μmol mol^–1^. The relative humidity was kept at 60% and the temperature at 25°C in the leaf chamber. Air flow rate was set at 750 μmol s^–1^ and air temperature was recorded automatically by the instrument.

Chlorophyll fluorescence and P700 parameters were measured simultaneously by Dual-PAM-100 system (Walz, Effeltrich, Germany), using the automated “Induction Curve” script provided by the software. Prior to measurements, all plants were in the dark for more than 2 h, and fluorescence induced curve (Slow Kinetics) was determined in “Fluo+P700” mode. Then the kinetics of Chl fluorescence induction and P700 oxidation were recorded simultaneously by the instrument. The light-adapted photosynthetic parameters were recorded after exposure to different light intensities (1445, 1105, 819, 592, 418, 206, 69, 36, 13 μmol photons m^–2^ s^–1^) for 240 s. The Chl fluorescence parameters were calculated as described in Huang et al. (2017): *Fv/Fm* = (*Fm-Fo*)*/Fm*, *Y*(*II*) = (*Fm’*-*Fs*)/*Fm’*, *Y*(*NPQ*) = *Fs/Fm’*-*Fs/Fm*, *Y*(*NO*) = *Fs*/*Fm*, where *Fm* and *Fm’* represent the maximum fluorescence after dark-adapted and light-adapted, respectively, and *Fo* and *Fo’* represent the minimum fluorescence in the dark-adapted state and light-adapted state, respectively. *Fo* and *Fm* were determined after dark adaptation for 2 h. *Fs* is the light-adapted steady-state fluorescence.

The P700 signals (*P*) may vary between a minimal (P700 fully reduced) and a maximal level (P700 fully oxidized). P700^+^ oxidation was monitored by absorbance changes in the near-infrared (830–875 nm). *P*_m_ indicated the maximal P700 signal observed upon full oxidation and was used to estimate the PSI activity, it was determined with application of a saturation pulse (240 ms and 582 μmol photons m^–2^ s^–1^) after pre-illumination with far-red light. *Y*(*NA*), the quantum yield of non-photochemical energy dissipation due to acceptor-side limitation, was calculated according to *Y*(*NA*) = (*P*_m_−*P_m_’*)/*P*_m_. The *Pm’*, similarly to *Pm*, indicated the maximal P700 signal induced by combined actinic illumination plus saturation pulse (240 ms and 582 μmol photons m^–2^ s^–1^). *Y*(*I*) indicated photochemical quantum yield of PSI and was estimated according to *Y*(*I*) = (*P_m_’*−*P*)*/P_m_*; *Y*(*ND*) indicated quantum yield of non-photochemical energy dissipation due to donor side limitation in PS I and was calculated by *Y*(*ND*) = *P*/*Pm*.

Photosynthetic electron flow through PSI and PSII was calculated as: ETRII = Y(II) × PPFD × 0.84 × 0.5, ETRI = Y(I) × PPFD × 0.84 × 0.5 (Yamori et al., 2016). The value of CEF was estimated as ETRI-ETRII (Huang et al., 2017). Chl fluorescence imaging of treated leaves was carried out at room temperature with an imaging PAM (ImagingWinGigE, Walz, Effeltrich, Germany) after dark conditioning for 1 h, according to procedures described by Xia et al. (2009).

The values of rETRmax and I_k_ were estimated using the empirical equation of rapid light curve (RLC): P=PAR/(αPAR^2^ + *bPAR* + *c*) proposed by Eilers and Peeters (1988). α = 1/*c*, $\mathrm{rETRmax}=\frac{1}{b+2\,\sqrt{a\times c}}$, ${\text{I}}_{\text{k}}=\frac{c}{b+2\,\sqrt{a\times c}}$. Where PAR is photosynthetically active radiation measured in μmol quanta m^–2^ s^–1^, *α* is light limiting region, rETRmax is maximum photosynthetic capacity, and I_k_ is minimum saturating irradiance and reflects tolerance ability of photosystems to high light intensity.

The electrochromic pigment shift (ECS) signal was monitored as the absorbance change at 515 nm using a Dual-PAM-100 (Walz, Effeltrich, Germany) equipped with a P515/535 emitter-detector module (Walz). Alfalfa seedlings were dark-adapted for 1 h prior to ECS signal was detected. The ECS signal was obtained after 240 s of illumination at 582 μmol photons m^–2^ s^–1^ actinic light (AL) intensities; afterward, the ECS decay was measured by switching off the AL for 150 s. Subsequently, ECS dark interval relaxation kinetics (DIRK_ECS_) was analyzed according to Schreiber and Klughammer (2008) and Huang et al. (2017). Total *pmf* was estimated from the total amplitude of the rapid decay of the ECS signal during the dark pulse. The slow relaxation of the ECS signal reflects the relaxation of the proton gradient (ΔpH) across the thylakoid membranes. The time constant of the first-orderECS relaxation (τECS) is inversely proportional to the proton conductivity (g_H_^+^) of the thylakoid membrane through the ATP synthase. Therefore, g_H_^+^ was estimated as the inverse of the decay time constant [1/τECS].

For each treatment, at least 15 leaves from different seedlings were used for above measurements of Chl fluorescence and P700 parameters.

### RuBisCO Activity and H^+^-ATPase Activity

The activities of RuBisCO and plasma membrane H^+^-ATPase were measured using commercial enzyme-linked immunoassay (ELISA) kits according to the manufacturer’s instruction (JL22709 and JL49554, Jianglai Biotech, China). Briefly, about 1 g fresh shoots from different treatments were homogenized in 9 mL cold phosphate buffer (PBS, 0.01 M, pH 7.40) on ice. The homogenates were then centrifuged at 5000 × *g* for 10 min at 4°C. The extract (supernatants) from plants captures the antibody and encapsulates the antibody onto the micro-pore plate to make the solid phase antibody. Then, the samples (RuBisCO or H^+^-ATPase) were added to the encapsulated micro-pore and combined with the labeled antibody to form the antibody antigen-enzyme-labeled antibody complex. After a thorough washing, the substrate TMB was added and colored. The color is positively correlated with the activities of RuBisCO or H^+^-ATPase. Finally, the absorbance was determined immediately at 450 nm, and the activities of these enzymes were calculated with a standard curve.

### Al Content

Al content was determined according to Wang et al. (2017) with minor modification. Briefly, fresh samples from treated plants were oven-dried for 72 h at 80 C, and then grounded to fine powder. A 0.5 g powder was digested in a 1:1 (v/v) nitric acid/hydrogen peroxide solution (HNO_3_/H_2_O_2_). Al content was then determined using an inductively coupled plasma emission spectrometer (ICP-AES; Iris Advantage 1000, Jarrell Ash Corp., Franklin, MA, United States).

### Indole-3-Acetic Acid (IAA) Content

Indole-3-acetic acid was extracted and purified according to the method described by Wang S. Y. et al. (2016). Briefly, 0.5 g fresh samples from different treatments were ground in liquid nitrogen, and then 5 mL pre-cooled 80% methanol, which contained 10 mg L^–1^ BHT (w/v), was added to the sample. The extraction was conducted at −20°C for overnight, solids were then separated by centrifugation with 20,000 *g* 15 min, and re-extracted for 30 min in an additional 5 mL of the same extraction solution. Subsequently, the supernatants were concentrated to 2.0 mL and passed through a Sep-Pak Plus C18 cartridge (SepPak Plus, Waters, United States). After washing with 3 mL 20% methanol containing 1% (v/v) acetic acid, the cartridges were eluted with 1 mL pure methanol for HPLC analysis, which was performed on a Shimadzu LC-10A HPLC (Shimadzu, Kyoto, Japan) system equipped with an SPD-10Avp detector.

### Contents of NADP^+^ and NADPH

The contents of NADP^+^ and NADPH were determined according to the manufacturer’s instruction. Briefly, about 0.1 g fresh shoots from different treatments were ground into homogenate with 1 mL acidic extracting solution (for NADP^+^) or 1 mL alkaline extracting solution (for NADPH) in a mortar on ice. The homogenate was then transferred to a 1.5 mL Eppendorf tube and immersed in a water-bath at 95°C for 5 min, quickly cooled in ice bath, and centrifuged at 10,000 × *g* for 10 min at 4°C. The 500 μL supernatant was collected in a new tube and another 500 μL alkaline extracting solution (for NADP^+^) or 500 μL acidic extracting solution (for NADPH) were added to neutralize, then mixed, and centrifuged at 10,000 × *g* for 10 min at 4°C. The supernatant was collected for analysis according to the manufacturer’s instruction (Cominbio, Suzhou, China).

### Contents of Superoxide Anion (O_2_^–^)

The content of O_2_^–^ was measured according to the manufacturer’s instruction (SA-1-G, Cominbio, Suzhou, China). In this experiment, fresh samples were used for extraction and all operations were conducted at low temperature (4°C) or in an ice bath. The absorbance at 530 nm was measured with a microplate reader (synergy2, BioTek, United States).

### Statistical Analysis

All above treatments were repeated three times, and the data were assessed from the results of three independent experiments. The main effect of treatments was calculated by analysis of variance (ANOVA) using SAS 9.0 (SAS Institute Inc., Cary, NC, United States). Treatment differences were tested using a mean separation test named least significant difference (LSD) at *P* ≤ 0.05 level.

## Results

### Growth Rate of Alfalfa Seedlings

**FIGURE 1:**
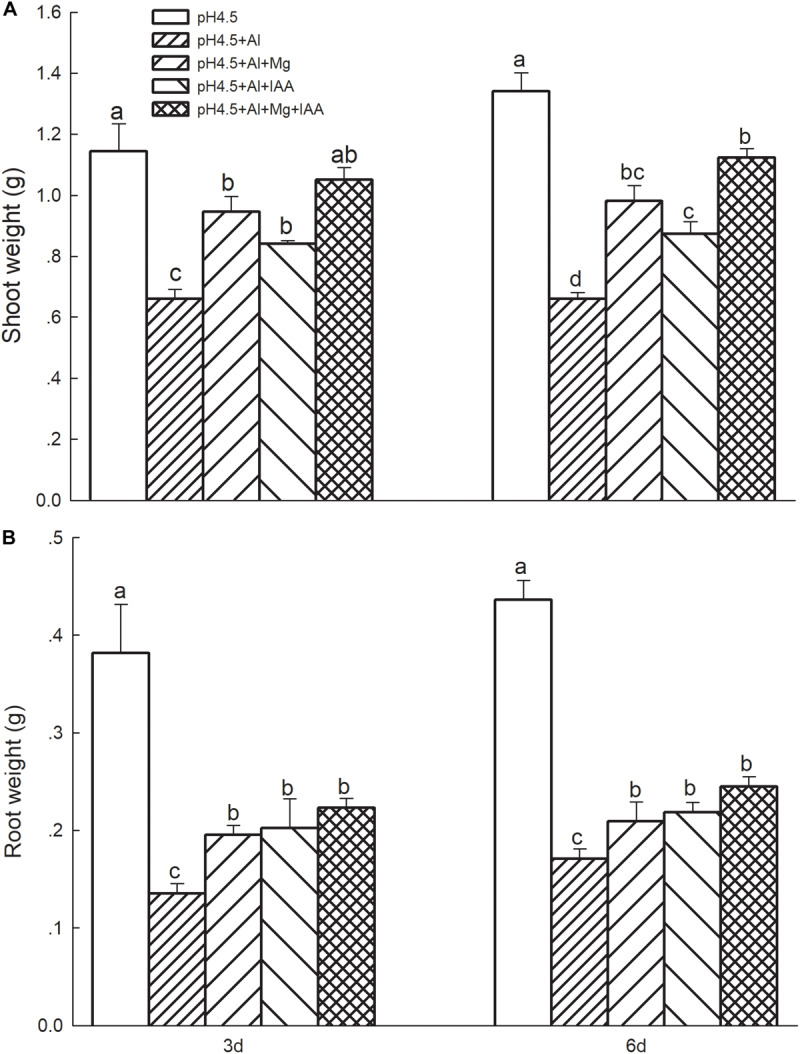
Shoot weight **(A)** and root weight **(B)** of Al stressed alfalfa seedlings with apical buds grown in 1.5 mM Ca(NO_3_)_2_ medium (pH 4.5) containing 0 μM AlCl_3_ (pH4.5), 100 μM AlCl_3_ (pH4.5+Al), 100 μM AlCl_3_ and 50 μM MgCl_2_ (pH4.5+Al+Mg), 100 μM AlCl_3_ and 6 mg L^–1^ IAA (foliar spray) (pH4.5+Al+IAA), or 100 μM AlCl_3_ and 50 μM MgCl_2_ and 6 mg L^–1^ IAA (foliar spray) (pH4.5+Al+Mg+IAA) at the third and sixth days after the initiation of treatments. Data are means ± SE of three replicates from three independent experiments. Bars with different letters indicate significant difference at *P* < 0.05 (least significant difference test).

Al stress significantly decreased fresh weights of shoots (Figure 1A) and roots (Figure 1B) compared with control treatment. Application of Mg and IAA significantly alleviated the Al-induced inhibition of growth, and the weights of shoots and roots were significantly higher under Mg and IAA application either alone or combination than that under Al treatments alone on days 3 and 6. Meanwhile, the weights were higher in the combined application of Mg and IAA than Mg or IAA application alone.

### IAA Contents and H^+^-ATPase Activity in Shoots

**FIGURE 2:**
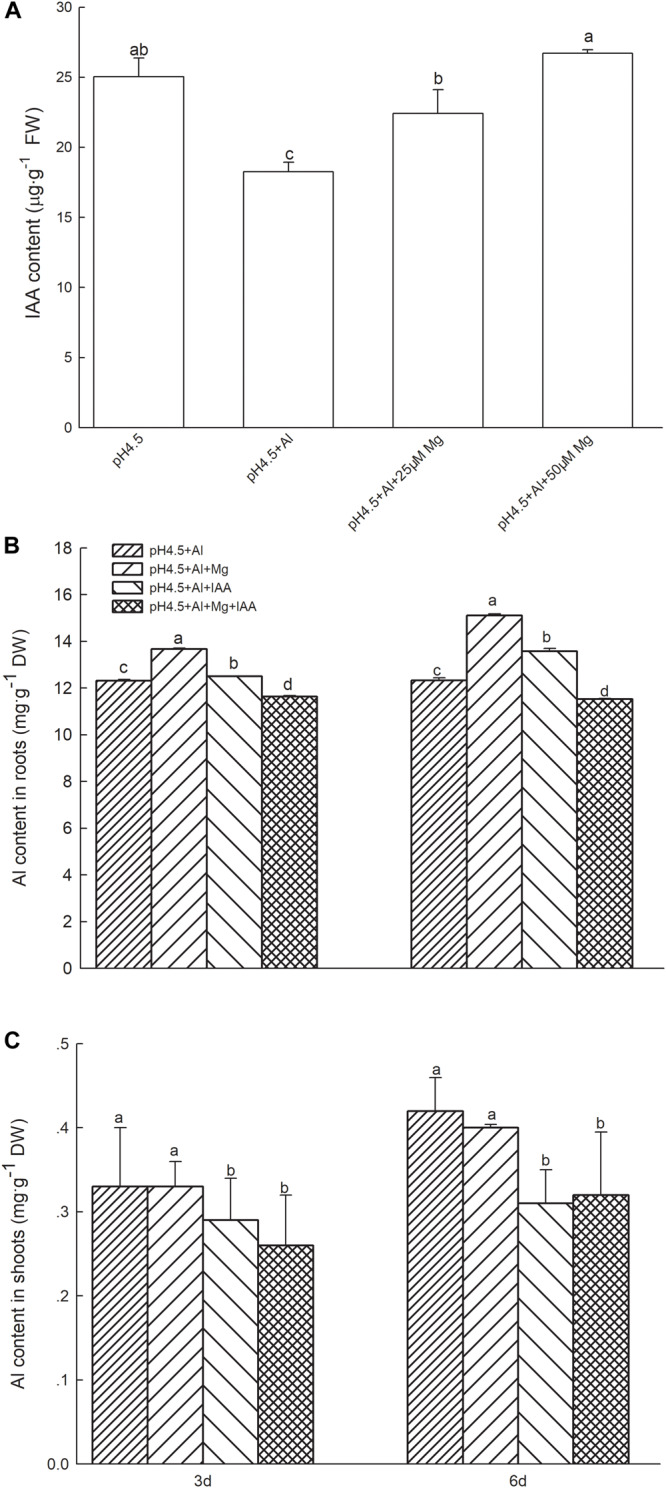
IAA and Al contents in Al stressed alfalfa seedlings. **(A)** IAA contents in shoots of alfalfa seedlings with apical buds grown in 1.5 mM Ca(NO_3_)_2_ medium (pH 4.5) containing 0 μM AlCl_3_ (pH4.5), 100 μM AlCl_3_ (pH4.5+Al), 100 μM AlCl_3_ and 25 μM MgCl_2_ (pH4.5+Al+25 μM Mg), and 100 μM AlCl_3_ and 50 μM MgCl_2_ at the third day after the initiation of treatments. Al contents in roots **(B)** and shoots **(C)** of alfalfa seedlings with apical buds grown in1.5 mM Ca(NO_3_)_2_ medium (pH 4.5) containing 0 μM AlCl_3_ (pH4.5), 100 μM AlCl_3_ (pH4.5+Al), 100 μM AlCl_3_ and 50 μM MgCl_2_ (pH4.5+Al+Mg), 100 μM AlCl_3_ and 6 mg L^–1^ IAA (foliar spray) (pH4.5+Al+IAA), or 100 μM AlCl_3_ and 50 μM MgCl_2_ and 6 mgL^–1^ IAA (foliar spray) (pH4.5+Al+Mg+IAA) at the third and sixth days after the initiation of treatments. Data are means ± SE of three replicates from three independent experiments. Bars with different letters indicate significant difference at *P* < 0.05 (least significant difference test).

Indole-3-acetic acid content in shoots of alfalfa were 37.0% lower in Al treatment than in control treatment on day 3, but addition of 25 and 50 μM Mg to the nutrient solution with 100 μM Al significantly increased the IAA contents by 22.6 and 46.2%, respectively, in comparison with Al treatment alone (Figure 2A).

**FIGURE 3:**
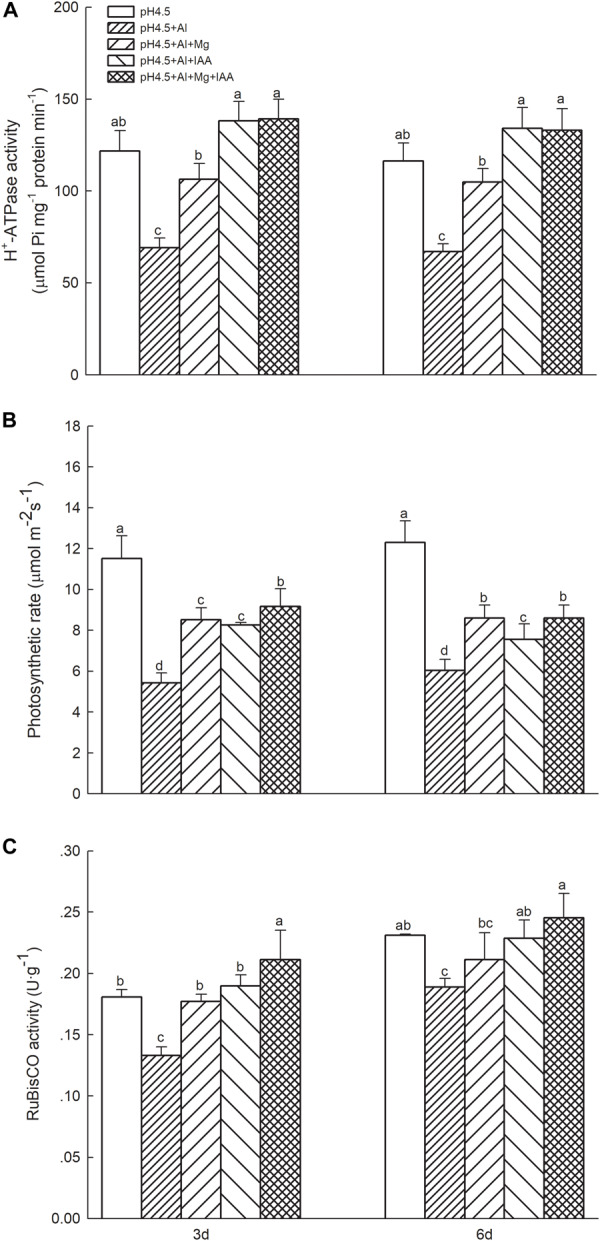
H^+^-ATPase activity, net photosynthetic rate, and RuBisCO activity in Al stressed alfalfa seedlings. **(A)** H^+^-ATPase activity in shoots of alfalfa seedlings with apical buds grown in 1.5 mM Ca(NO_3_)_2_ medium (pH 4.5) containing 0 μM AlCl_3_ (pH4.5), 100 μM AlCl_3_ (pH4.5+Al), 100 μM AlCl_3_ and 25 μM MgCl_2_ (pH4.5+Al+25 μM Mg), and 100 μM AlCl_3_ and 50 μM MgCl_2_ at the third day after the initiation of treatments. **(B)** Net photosynthetic rate and **(C)** RuBisCO activities in leaves of alfalfa seedlings with apical buds grown in 1.5 mM Ca(NO_3_)_2_ medium (pH 4.5) containing 0 μM AlCl_3_ (pH4.5), 100 μM AlCl_3_ (pH4.5+Al), 100 μM AlCl_3_ and 50 μM MgCl_2_ (pH4.5+Al+Mg), 100 μM AlCl_3_ and 6 mg L^–1^ IAA (foliar spray) (pH4.5+Al+IAA), or 100 μM AlCl_3_ and 50 μM MgCl_2_ and 6 mg L^–1^ IAA (foliar spray) (pH4.5+Al+Mg+IAA) at the third and sixth days after the initiation of treatments. Data are means ± SE of three replicates from three independent experiments. Bars with different letters indicate significant difference at *P* < 0.05 (least significant difference test).

Excess Al significantly decreased folia H^+^-ATPase activity in comparison with control treatments, while application of Mg and IAA either alone or combination significantly increased H^+^-ATPase activities in Al-stressed alfalfa seedlings on days 3 and 6. The increase was higher in IAA treatments than in Mg treatments (Figure 3A).

### Al Contents in Roots and Shoots

Application of Mg or IAA significantly increased Al contents in roots compared with Al treatment alone, and the Al content was higher in Mg addition than IAA application (Figure 2B). The combined application of Mg and IAA, however, greatly decreased Al absorption, and its Al contents were significantly lower than Al treatments either alone or combined with Mg or IAA application.

Application of Mg did not affect Al transfer from roots to shoots, and Al contents in shoots were not significant different between Al treatments and Mg treatments. However, application of IAA greatly decreased Al contents in shoots compared with Al treatments with or without Mg addition (Figure 2C). These results were consistent in 3 and 6 days.

### Pigment Contents, Net Photosynthetic Rates, and Rubisco Activities

Excess Al decreased the contents of Chl a (Supplementary Figure S2a) and Chl b (Supplementary Figure S2b), especially on 6 days, but application of Mg and IAA either alone or combination significantly increased contents of the two pigments under Al stress. The Pn decreased by 54.36 and 35.45% on 3 and 6 days, respectively, under Al treatments in comparison with control treatments, but application of Mg and IAA either alone or combination significantly increased Pn of Al-stressed seedlings, and the Pn was higher in Mg+IAA treatments than Mg or IAA application alone (Figure 3B). Foliar rubisco activities significantly decreased under Al treatments in comparison with control treatments, but application of Mg or IAA significantly increased rubisco activities of Al-stressed alfalfa seedlings on days 3 and 6 (Figure 3C), and the rubisco activities were higher in combined application of Mg and IAA than Mg or IAA application alone.

### Chlorophyll Fluorescence Parameters of PSI and PSII

**FIGURE 4:**
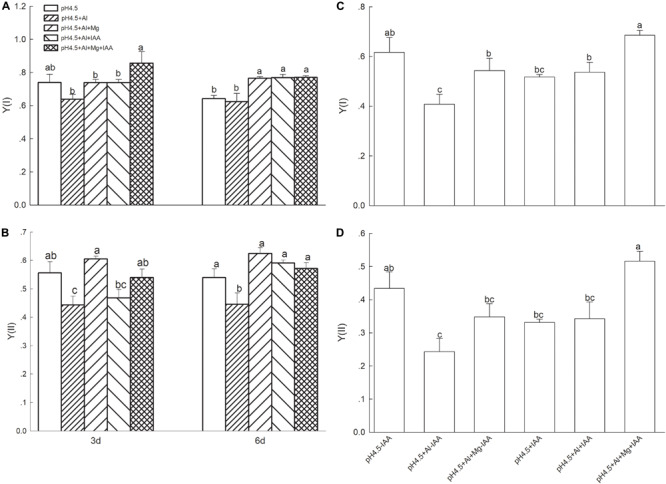
Light intensity dependence of photosynthetic quantum yields of PSI [Y(I)] and PSII [(Y(II)] in leaves of Al stressed alfalfa seedlings with or without apical buds. Five treatments in the seedlings with apical buds are as Figure 1, and seedlings without apical buds are grown in 1.5 mM Ca(NO3)_2_ medium (pH 4.5) and treated with or without spraying IAA (pH4.5-IAA, pH4.5+IAA), 100 μM AlCl_3_ with or without spraying IAA (pH4.5+Al-IAA, pH4.5+Al+IAA), and 100 μM AlCl_3_ and 50 μM MgCl_2_ with or without spraying IAA (pH4.5+Al+Mg-IAA, pH4.5+Al+Mg+IAA). The Y(I) **(A)** and Y(II) **(B)** were estimated from seedlings with apical buds, and Y(I) **(C)** and Y(II) **(D)** were estimated from seedlings without apical buds at the third day after the initiation of treatments. Data are means ± SE of three replicates. Bars with different letters indicate significant difference at *P* < 0.05 (least significant difference test).

Under Al stress, application of Mg or IAA significantly increased the effective quantum yields of PSI [Y(I)] on 6 days, while combined application of Mg and IAA increased it on days 3 and 6 (Figure 4A). Effective quantum yields of PSII [Y(II)] were significantly lower in Al treatment alone than that in control and in Al treatments with Mg and IAA application either alone or combine on days 3 and 6 (Figure 4B). Application of Mg and IAA either alone or combine significantly decreased Y(ND), Y(NA), and Y(NPQ) on day 6 under Al stress (Supplementary Figure S3).

**FIGURE 5:**
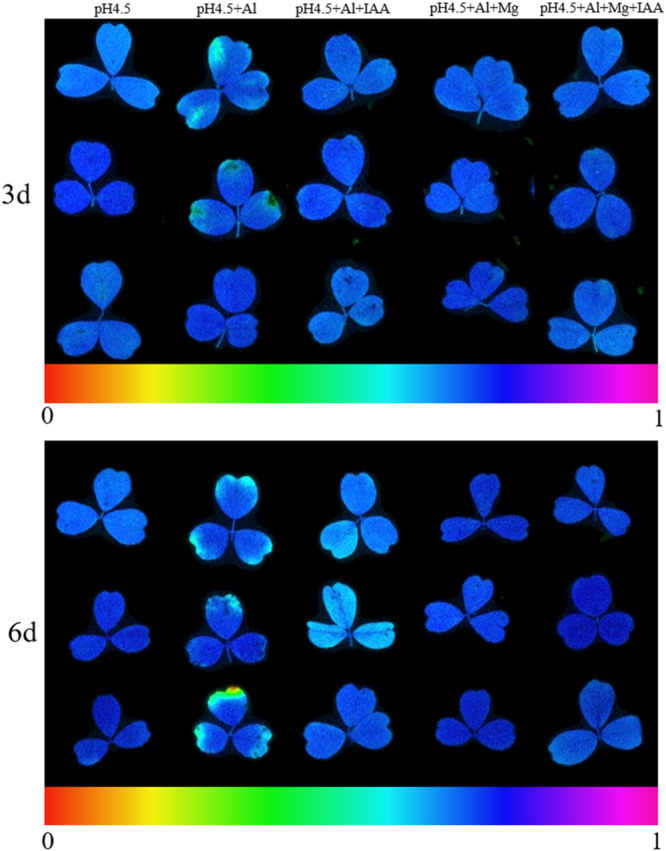
Images of chlorophyll fluorescence in leaves of Al stressed alfalfa seedlings with apical buds grown in 1.5 mM Ca(NO_3_)_2_ medium (pH 4.5) containing 0 μM AlCl_3_ (pH4.5), 100 μM AlCl_3_ (pH4.5+Al), 100 μM AlCl_3_ and 50 μM MgCl_2_ (pH4.5+Al+Mg), 100 μM AlCl_3_ and 6 mg L^–1^ IAA (foliar spray) (pH4.5+Al+IAA), or 100 μM AlCl_3_ and 50 μM MgCl_2_ and 6 mgL^–1^ IAA (foliar spray) (pH4.5+Al+Mg+IAA) at the third and sixth days after the initiation of treatments.

PSII function can be assessed by Fv/Fm. The values of *Fv/Fm* was lower in Al treatments than control treatment (Supplementary Figure S4), and Chl fluorescence image also showed a decline of Fv/Fm in Al treatments, in which the number and area of yellow-green spots in leaves were larger in Al treatment alone than in other four treatments (Figure 5). The combined application of Mg and IAA had most significant effects on alleviating Al induced damage on primary photochemistry.

After removing apical buds of alfalfa seedlings, the quantum yields of Y(I) and Y(II) significantly decreased under Al stress compared with control treatment under none IAA application, but they were no significant difference between Al and control treatments under IAA application (Figures 4C,D). Mg addition significantly increased Y(I) and Y(II) compared with Al treatment under IAA application, as well as Y(I) under none IAA application.

### Light Intensity Dependence of Photosynthetic Electron Flow

**FIGURE 6:**
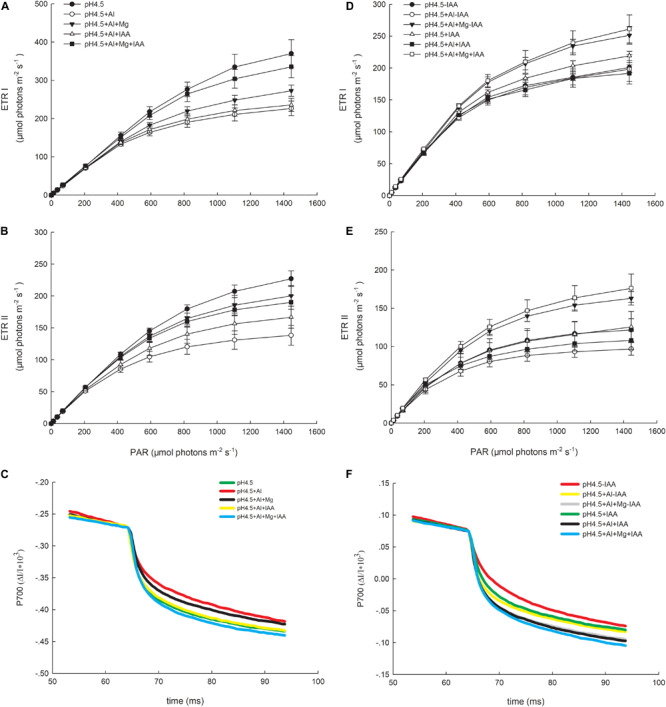
Light intensity dependence of the photosynthetic electron flow through PSI (ETRI) and PSII (ETRII), and P700^+^ reduction curve in leaves of Al stressed alfalfa seedlings with or without apical buds. Five treatments in the seedlings with apical buds are as Figure 1, and seedlings without apical buds are grown in 1.5 mM Ca(NO3)_2_ medium (pH 4.5) and treated with or without spraying IAA (pH4.5-IAA, pH4.5+IAA), 100 μM AlCl_3_ with or without spraying IAA (pH4.5+Al-IAA, pH4.5+Al+IAA), and 100 μM AlCl_3_ and 50 μM MgCl_2_ with or without spraying IAA (pH4.5+Al+Mg-IAA, pH4.5+Al+Mg+IAA). The ETRI **(A)**, ETRII **(B)**, and P700^+^ reduction curve **(C)** were estimated from seedlings with apical buds, and ETRI **(D)**, ETRII **(E)**, and P700^+^ reduction curve **(F)** were estimated from seedlings without apical buds at the third day after the initiation of treatments.

Photosynthetic ETRs through PSI (ETRI) and PSII (ETRII) were strongly inhibited by Al stress compared with control (Figure 6). Under a high light intensity (1445 μmol photons m^–2^ s^–1^), ETRI values in the order of control (pH4.5), pH4.5+Al, pH4.5+Al+Mg, pH4.5+Al+IAA, and pH4.5+Al+Mg+IAA were 369.9, 226.6, 272.8, 235.7, and 335.5 μmol electrons m^–2^ s^–1^, respectively (Figure 6A), and ETRII values were 227.1, 138.4, 200.4, 166.7, and 190.2 μmol electrons m^–2^ s^–1^, respectively (Figure 6B). Meanwhile, values of maximum photosynthetic capacity (rETRmax) in PSI and PSII were also inhibited by Al stress compared with control on 3 days, but application of Mg and IAA alleviated the negative effects on days 3 and 6 (Table 1).

**TABLE 1:** Effects of Mg and IAA on maximum relative electron transport rate (rETRmax) and minimum saturating irradiance (I_k_) in PSI and PSII under Al stress.

			**pH4.5**	**pH4.5+Al**	**pH4.5+Al+Mg**	**pH4.5+Al+IAA**	**pH4.5+Al+Mg+IAA**
3 days	PSI	rETRmax	250.7 ± 5.5a	198.4 ± 15.1d	301.63 ± 10.8bc	261.6 ± 16.4c	318.8 ± 13.5ab
		I_k_	953.5 ± 40.3a	543.4 ± 72.1c	740.9 ± 13.9b	642.9 ± 25.5bc	917.7 ± 77.1a
	PSII	rETRmax	249.7 ± 20.5a	115.5 ± 4.1d	224.1 ± 7.0ab	182.6 ± 14.3c	207.2 ± 8.3bc
		I_k_	827.7 ± 50.1a	405.7 ± 38.1c	710.4 ± 32.1b	634.3 ± 25.9b	662.4 ± 38.6b
6 days	PSI	rETRmax	247.5 ± 7.5ab	220.5 ± 23.8b	286.8 ± 4.4a	296.7 ± 28.1a	305.6 ± 16.1a
		I_k_	679.4 ± 59.4bc	657.1 ± 66.8c	768.8 ± 44.1a	758.2 ± 51.5a	727.7 ± 32.5ab
	PSII	rETRmax	151.8 ± 13.7b	152.7 ± 20.3b	223.2 ± 6.1a	217.4 ± 14.6a	212.2 ± 7.2a
		I_k_	655.9 ± 76.8ab	507.1 ± 91.5b	718.6 ± 35.1a	711.7 ± 52.6a	690.9 ± 15.3a

*Data are the means ± SE. Values followed by different letters are significantly different at *p* ≤ 0.05.*

P700^+^ reduction curve reflects the releasing rate of electron from oxidized P700, and the higher the initial slope of the curve, the higher the releasing rate of electron or the higher the amount of active population of P700 is. The initial slopes of the curve were lowest in Al treatment alone, and highest in combined application of Mg and IAA (Figure 6C).

The values of I_k_ of PSI and PSII decreased in Al treatments alone compared with control treatments, but application of Mg and IAA either alone or combine increased the I_k_ values under Al stress (Table 1).

After removing apical buds of alfalfa seedlings, excess Al greatly inhibited ETRI under IAA application and ETRII under both application and none application of IAA compared with control treatments (Figures 6D,E). Mg addition greatly increased ETRI and ETRII compared with control and Al treatment under both application and none application of IAA. Meanwhile, ETRI and ETRII were higher under IAA application than none IAA application with or without Al and Mg addition except for ETRI under Al stress alone. The initial slopes of the P700^+^ reduction curve were higher under IAA application than none IAA application, and the highest initial slope was in Mg addition under IAA application (Figure 6F).

### Cyclic Electron Flow

**FIGURE 7:**
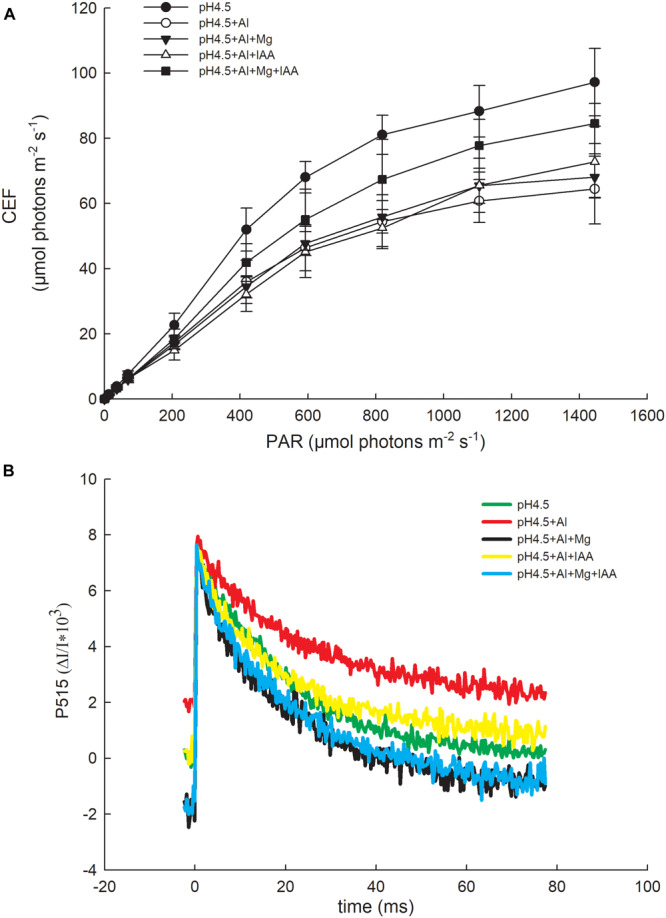
Cyclic electron flow around PSI (CEF) **(A)** and changes of P515 signal **(B)** in leaves of Al stressed alfalfa seedlings with apical buds grown in 1.5 mM Ca(NO_3_)_2_ medium (pH 4.5) containing 0 μM AlCl_3_ (pH4.5), 100 μM AlCl_3_ (pH4.5+Al), 100 μM AlCl_3_ and 50 μM MgCl_2_ (pH4.5+Al+Mg), 100 μM AlCl_3_ and 6 mg L^–1^ IAA (foliar spray) (pH4.5+Al+IAA), or 100 μM AlCl_3_ and 50 μM MgCl_2_ and 6 mgL^–1^ IAA (foliar spray) (pH4.5+Al+Mg+IAA) at the third day after the initiation of treatments.

The light response change of cyclic electron flow (CEF) around PSI increased after Mg and IAA applications compared with Al treatment alone. Under a high light intensity (1445 μmol photons m^–2^ s^–1^), the values of CEF under control (pH4.5), pH4.5+Al, pH4.5+Al+Mg, pH4.5+Al+IAA, and pH4.5+Al+Mg+IAA were 97.2, 64.4, 68.1, 72.8, and 84.5 μmol photons m^–2^ s^–1^, respectively (Figure 7A).

### Proton Motive Force and Thylakoid Proton Conductivity

**FIGURE 8:**
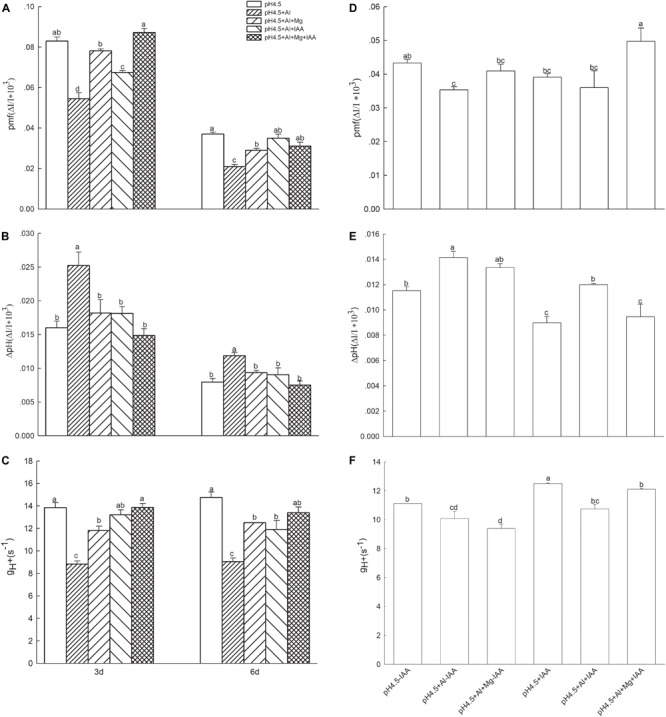
Parameters derived from the dark-interval relaxation kinetic of ECS (DIRK_ECS_) in Al stressed seedlings with or without apical buds. Five treatments in the seedlings with apical buds are as Figure 1, and seedlings without apical buds are grown in 1.5 mM Ca(NO3)_2_ medium (pH 4.5) and treated with or without spraying IAA (pH4.5-IAA, pH4.5+IAA), 100 μM AlCl_3_ with or without spraying IAA (pH4.5+Al-IAA, pH4.5+Al+IAA), and 100 μM AlCl_3_ and 50 μM MgCl_2_ with or without spraying IAA (pH4.5+Al+Mg-IAA, pH4.5+Al+Mg+IAA). Total proton motive force (*pmf*) **(A)**, the proton gradient (ΔpH) **(B)**, and the thylakoid proton conductivity (g_H_^+^) **(C)** were estimated from seedlings with apical buds, and *pmf* **(D)**, ΔpH **(E)**, and g_H_^+^ **(F)** were estimated from seedlings without apical buds at the third day after the initiation of treatments. Bars with different letters indicate significant difference at *P* < 0.05 (least significant difference test).

The light intensity dependence of DIRK_ECS_ kinetics is shown in Figure 8. At light intensity of 582 μmol photons m^–2^ s^–1^, Al stress significantly decreased the total *pmf*, but application of Mg and IAA either alone or combine significantly increased *pmf* under Al stress on days 3 and 6 (Figure 8A). The values of proton gradient (ΔpH*_pmf_*) were significantly higher in Al treatment alone than that in the treatments of control (pH4.5), Al+Mg, Al+IAA and Al+Mg+IAA on days 3 and 6 (Figure 8B). Al stress decreased the proton conductivity (g_H_^+^) of the thylakoid membrane, and application of Mg and IAA either alone or combine elevated g_H_^+^ under Al stress (Figure 8C). The combined application of Mg and IAA had higher effect on increasing *pmf* and g_H_^+^, and decreasing ΔpH*_pmf_* than Mg or IAA application alone.

After removing apical buds of alfalfa seedlings, excess Al significantly decreased *pmf* under none IAA application, but did not significantly affect *pmf* under IAA application. Mg addition significantly increased *pmf* under IAA application, but did not significantly affect *pmf* under none IAA application compared with control and Al stress treatments (Figure 8D). Excess Al significantly increased ΔpH*_pmf_* in both application and none application of IAA, but Mg addition significantly decreased ΔpH*_pmf_* under IAA application, and the ΔpH*_pmf_* was lower under IAA application than none IAA application after Mg addition (Figure 8E). Excess Al significantly decreased proton conductivity (g_H_^+^) of the thylakoid membrane under both application and none application of IAA, but Mg addition significantly increased g_H_^+^ under IAA application (Figure 8F).

### Activation State of ATP Synthase

Changes in P515-535 signal reflect the active state of the ATP synthase, the integrity of the thylakoid membrane, and the membrane potential difference across the thylakoid membrane (Schreiber and Klughammer, 2008). The higher the initial slope in the fast decline phase of P515 curve, the higher the activation state of the ATP synthase is. The decline phase of the initial slope was lowest in Al treatment alone, and was highest in the treatments of Mg and Mg+IAA, indicating that the activities of ATP synthase were increased by application of Mg and Mg+IAA (Figure 7B).

### Contents of NADP^+^, NADPH, and O_2_^–^

**FIGURE 9:**
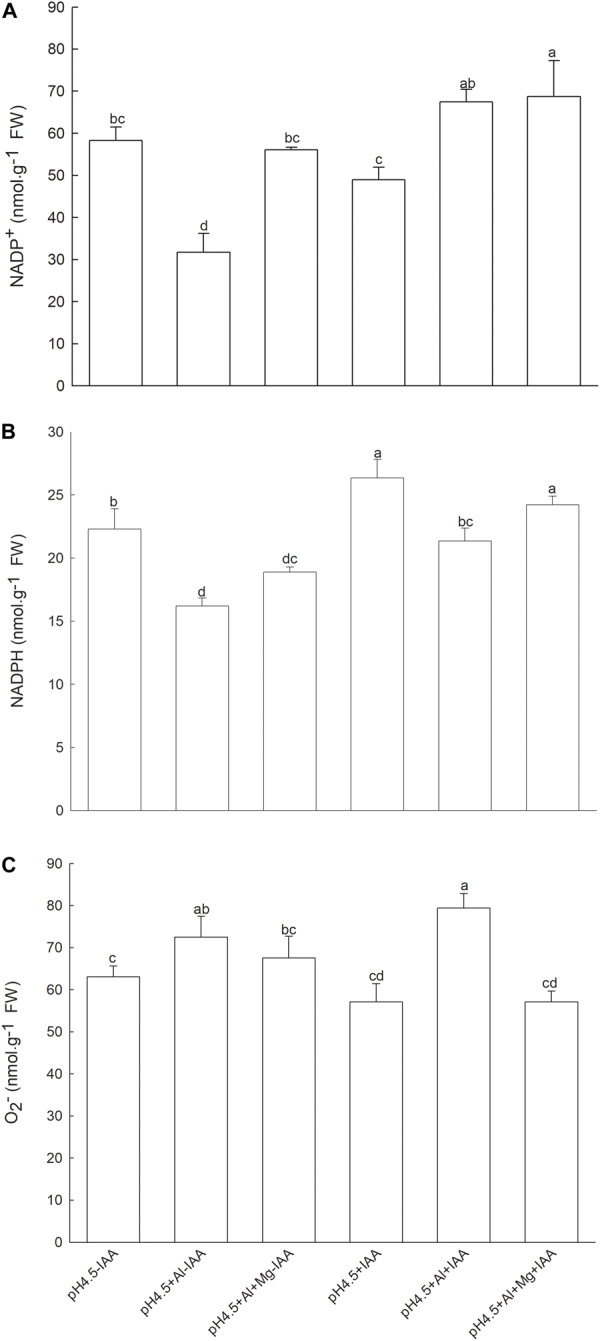
Contents of NADP^+^, NADPH and superoxide anion (O_2_^–^) in leaves of Al stressed alfalfa seedlings without apical buds. Seedlings grown in 1.5 mM Ca(NO_3_)_2_ medium (pH 4.5) were treated with or without spraying IAA (pH4.5-IAA, pH4.5+IAA), 100 μM AlCl_3_ with or without spraying IAA (pH4.5+Al-IAA, pH4.5+Al+IAA), and 100 μM AlCl_3_ and 50 μM MgCl_2_ with or without spraying IAA (pH4.5+Al+Mg-IAA, pH4.5+Al+Mg+IAA). NADP^+^ **(A)**, NADPH **(B)**, and O_2_^–^ **(C)** were measured at the third day after the initiation of treatments. Data are means ± SE of three replicates from three independent experiments. Bars with different letters indicate significant difference at *P* < 0.05 (least significant difference test).

After removing apical buds of alfalfa seedlings, contents of NADP^+^ in Al-stressed seedlings were significantly decreased under none IAA application, but were significantly increased under IAA application compared with control treatments. Mg addition significantly increased the NADP^+^ contents under none IAA application (Figure 9A). Contents of NADPH were significantly decreased by Al stress under both application and none application of IAA, but Mg addition significantly increased NADPH content under IAA application, and the NADPH content was higher under IAA application than none IAA application (Figure 9B). Contents of O_2_^–^ were significantly increased by Al stress compared with control treatments under both application and none application of IAA, but were significantly decreased by Mg addition under IAA application (Figure 9C).

## Discussion

Photosynthesis is a key mechanism for providing energy and organic molecules for plant growth and development. Photosynthesis incorporates light energy into ATP and NADPH via a multi-step process including photosystems (Nouri et al., 2015). Excess aluminum has been reported to inhibit plant growth, reduces Pn of *Eucalyptus* (Yang et al., 2015), damages PSII, and decreases its electron transport in *Citrus reshni Hort.* ex Tanaka (Chen et al., 2005; Li et al., 2012). In the present study, excess Al significantly decreased Pn and contents of Chl a and Chl b, which were positively related to the reductions of Fv/Fm, Y(II), ETRII, ETRI, and rETRmax of PSI and PSII, indicating that Al-induced decreases in Pn were greatly attributed to the inhibition of photosystems, rather than stoma limitation.

Magnesium is a key constituent of Chl molecules and plays an essential role in Chl formation and in activating photosystems in plants (Nèjia et al., 2015). Mg deficiency has been found to increase Chl degradation (Jin et al., 2016) and inhibits photosystems of plants (Nèjia et al., 2015). Al can compete to binding sites with Mg on the plasma membrane of roots, which interferes with Mg uptake and transport, and aggravates Mg deficiency (Thomas et al., 2004; An et al., 2014). A positive correlation between Y(II) and Mg concentration in the leaves of *Citrus reticulata* is verified under Al stress (Jin et al., 2016). Similarly, photoprotection for PSII and PSI under Al stress was observed after Mg application in the present study. Application of Mg increased the Chl contents (Chl a and Chl b) and values of saturating irradiances (I_k_) in PSI and PSII, indicating that Mg enhanced light use efficiency of PSI and PSII under Al stress, and consequently increased the effective quantum yields of Y(II) and Y(I) compared with excess Al treatment alone. These increases of effective quantum yields accounted for 61–62 and 74–76% of the total excitation energy in PSII and PSI, respectively, thereby effectively decreased the amount of non-photochemical energy dissipation. These would provide enough excited state energy to activate RCs of PSII and PSI; as a result, accelerated linear electron transfer from PSII to PSI and increased ETRII and ETRI.

PSI can function as a safe quencher of excitation energy when the electron flow to molecular oxygen is prevented. This prevention can occur either by limiting the electron transfer to PSI (Suorsa et al., 2012), by donating the electrons to alternative electron acceptors (Allahverdiyeva et al., 2013), or by decreasing the amount of active PSII centers to a level low enough to eliminate the capacity of photosynthetic machinery to produce excess electron (Tikkanen et al., 2014). Under Al stress, application of Mg greatly decreased the oxidized state P700 as seen from the evidence of lower level of Y(ND) (Supplementary Figure S3), and increased the amount of active population of P700 reflected by a faster decline phase of P700^+^ reduction curve (Figure 6C). These results indicated a nearly free outflow of electrons on the donor side of PSI, and a fast electron transfer through alternative electron acceptors of PSI, which led to higher levels of ETRI and rETRmax, and lower level of electron accumulation in PSI.

Many studies have confirmed the role of CEF in photoprotection of PSI and PSII (Suorsa et al., 2016; Yamori et al., 2016). CEF can reduce electron accumulation in the acceptor side of PSI by oxidizing the acceptor-side components of PSI, and recycling the electrons from PSI to PQ pool and Cyt b_6_f (Munekage et al., 2004; Tikkanen et al., 2015). In the present study, application of Mg increased CEF, which led to recycle excess electrons to Cyt b_6_f rather than to molecular oxygen (Figure 9C), decreased electron accumulation in PSI, and protected PSI against oxidative damage (Chaux et al., 2015). In addition, a lower dosage of Mg application (25 μM) on Al-stressed alfalfa seedlings greatly increased rETRmax and I_k_ of PSI and PSII, and the increases were higher in PSI than PSII (data not shown). Similarly, the increased degree of Y(II) was lower than Y(I) after Mg addition under Al stress. All the above results indicated that PSI was more sensitive to Mg deficiency than PSII, and Mg could alleviate Al-induced damages in PSI and PSII. Thus, although Mg application increased Al contents in roots, Pn still increased under Al stress. The result of increased Al content after Mg addition was inconsistent with previous study, in which Mg addition decreased Al content in root tips of rice bean by increasing citrate secretion (Yang et al., 2007). This might be variety specificity in citrate secretion. The citrate content in roots and citrate secretion from root tips of alfalfa are extremely low (An et al., 2014). The increased photosynthesis under relatively high Al content in roots is partly attributed to Mg-induced increase in IAA content, because IAA application and its interaction with Mg decreased Al transfer from roots to shoots (Figure 2C). In addition, IAA can decrease pectin methylesterase activity in alfalfa under Al stress, which reduces the number of functional groups with negative charge in cell wall, consequently decreases Al accumulation in pectin (Wang et al., 2017) and increases extendability of Al-stressed cell wall (Mikko and Aro, 2014; Guo et al., 2016). Thus, the plant growth was increased after application of Mg and IAA under Al stress.

As a systemic signal, auxin directly affects photosynthetic progress (Guo et al., 2016). In the present study, excess Al decreased foliar IAA content and Pn, but the decreases were significantly alleviated by Mg application, while exogenous application of IAA significantly increased Pn of Al-stressed alfalfa seedlings, indicating that IAA content in shoots is positively related to Pn under Al stress and plays an important role in alleviating Al induced damages on photosynthetic machinery. Kim et al. (2001) reported that an auxin-binding protein, ABP57, promoted the pumping activity of H^+^-ATPase in the presence of IAA, which, in turn, regulated H^+^ extrusion. Overexpression of *OsPIN2* ameliorates the Al inhibitory effect on basipetal auxin transport and increases H^+^ secretion from root tips (Wu et al., 2014). Our previous study also demonstrated that exogenous application of IAA increased H^+^ secretion from root tips of Al-stressed alfalfa seedlings (Wang et al., 2017). Through extruding protons, the electrochemical gradient (proton gradient, ΔpH) across the plasma membrane is generated, which is necessary to activate electron transfer, polar auxin transport, and cell elongation (Rober-Kleber et al., 2003; Wang P. et al., 2016).

Indole-3-acetic acid transport in cell is regulated by two structurally separated plasma membrane transport processes: PIN-FORMED (PIN) protein family of auxin transporters mediating IAA efflux, and PM H^+^-ATPase mediating H^+^ efflux (Wang P. et al., 2016; Zhang et al., 2017). IAA acts as a signal to increase CEF and ATP generation, which increases H^+^ transfer from lumen to stroma and decreases ΔpH between the thylakoid lumen and stroma (Guo et al., 2016). In the thylakoid membrane, ΔpH between the lumen and stroma is dependent on: (i) the accumulation of protons in the lumen from the water-splitting activity of PSII and from the electron transfer via Cyt b_6_f, including LEF, Q-cycle, and CEF; and (ii) the rate of proton efflux from the lumen (i.e., activity of the ATP synthase in releasing H^+^); and (iii) the Cyt b_6_f complex couples the electron transfer to proton transfer or the proton gradient-dependent control of electron transfer (Mikko and Aro, 2014). When net ATP synthesis is zero, pH in the lumen can decrease to as low as pH 5.2, a pH value at which the water-splitting activity and the reduction kinetics of P680^+^ start to slow down (Thomas and Anja, 2014). Thus, the effect of auxin on photosynthesis may relate to the H^+^ transfer in cells and H^+^ secretion from roots by activating H^+^-ATPase (Xu et al., 2012; Wu et al., 2014).

Based on the above results, we speculated that Mg induced alleviation of photosystem damage might be partly attributed to the increases in IAA synthesis and transport in alfalfa under Al stress, in which IAA, alone or interactive with Mg, regulated proton efflux (g_H_^+^) and the formation of ΔpH in thylakoid. Thus, we further studied the interactive effect of Mg and IAA on regulating ΔpH formation in thylakoid of Al-stressed alfalfa seedlings by exogenous application of IAA. The results showed that exogenous application of IAA, alone or combined with Mg, significantly increased *pmf*, CEF, PM H^+^-ATPase activity, and g_H_^+^ from lumen to stroma under Al stress, and decreased ΔpH*_pmf_* between lumen and stroma (Figures 7A, 8A–C). The combined application of Mg and IAA had the highest effects on the alleviation of photosystem damage, indicating an interaction of Mg and IAA on regulating the proton and electron transfer under Al stress. The generation of *pmf*, partly dependent on CEF, has two main roles: one is linked to ATP synthesis and balancing ATP/NADPH ratios, and the other is dependent on lumen acidification and favors photoprotection in PSI and PSII (Munekage et al., 2004). The interaction of Mg and IAA induced increase of *pmf* in Al-stressed seedlings activated ATP synthase located in thylakoid membrane (Figure 7B), which directly increased ATP synthesis, accompanied with H^+^ transfer from lumen to stroma. These would decrease ΔpH*_pmf_* between lumen and stroma. Meanwhile, the CEF-dependent *pmf* promoted g_H_^+^, associated with high H^+^-ATPase activity, further decreased the ΔpH*_pmf_*, and promoted proton efflux to stroma from lumen, to apoplastic space from internal cells and H^+^ secretion from root tips (Wang et al., 2017), and thus ameliorated H^+^ environment in cells under Al stress. The acidification of the thylakoid lumen controls photosynthetic electron transport by slowing PQ oxidation at the Cyt b_6_f, and activates the NPQ to dissipate excess excitation energy as heat in the harmless form to protect PSII (Suorsa et al., 2012; Tikkanen et al., 2014). The ΔpH*_pmf_* formation controls modulation of the PSII antenna light harvesting “switch.” The higher the ΔpH*_pmf_*, the slower the electron transfer rate from PSII to PSI, and the slower the energy transfer efficiency from LHCII to the photosystems (Mikko and Aro, 2014). Thus, the Y(I), Y(II), ETRI, and ETRII in the present study were significantly increased by exogenous application of IAA, especially for combination with Mg, compared with Al treatment alone.

**FIGURE 10:**
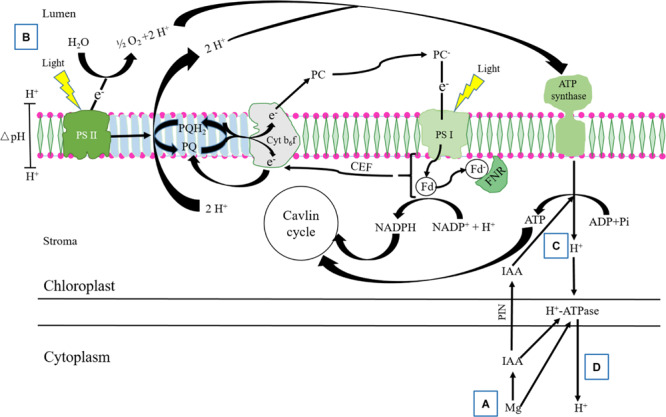
Mg alleviation of Al-induced photoinhibition via increasing IAA content and transport. Under Al stressed condition, **(A)** application of Mg increases IAA synthesis and transport in cells; **(B)** the accumulation of protons produced from water-splitting activity of PSII and from the electron transfer via Cyt b_6_f cause lumen acidification; **(C)** IAA promotes, on the one hand, CEF activity and H^+^-dependent ATP synthesis by activating ATP synthase in thylakoid accompanying with H^+^ transfer from lumen to stoma, leading to decrease ΔpH*_pmf_* between lumen and stroma. The increased CEF and decreased ΔpH*_pmf_* help to sustain electron transport from PSII to PSI and NADPH generation, increasing CO_2_ fixation under excess Al stress; **(D)** on the other hand, IAA and Mg increase PM H^+^-ATPase activity, which enhances H^+^ transfer from stoma to cytoplasm, further decreases the ΔpH*_pmf_* between the lumen and stroma, and balances H^+^ between intracellular and extracellular.

Our previous study showed that IAA was synthetized in apical buds of alfalfa, and the IAA concentration in root tips decreased to 11.4 and 10.3% of normal seedlings in control and Al treatments, respectively, after removing apical buds for 24 h (Wang S. Y. et al., 2016). Thus, we used the phenomenon of IAA natural decrease after removing apical buds of alfalfa seedlings to further test the interactive effect of Mg and IAA on regulating ΔpH formation in thylakoid under Al stress. In this study, the apical buds of alfalfa seedlings were removed and the seedlings were sprayed with water or IAA. Under none spray IAA, Mg addition did not significantly alleviate Al induced damage on photosystem, and the values of Y(II), *pmf*, ΔpH*_pmf_*, and g_H_^+^ were not significantly different compared with Al treatment alone, indicating that Mg did not affect proton conductivity and ΔpH*_pmf_* formation in the absence of IAA under Al stress. Exogenous spray IAA, however, significantly increased *pmf* and g_H_^+^, and decreased ΔpH*_pmf_* under Mg addition compared with Al treatment alone; as a result, Y(I), Y(II), ETRI, and ETRII significantly increased. These results clearly demonstrated that there were strong interactions between Mg and IAA on photoprotection in PSI and PSII under Al stress. Mg alleviating Al-induced photoinhibition in photosystems was greatly attributed to IAA participation via increasing *pmf* and PM H^+^-ATPase activity, and decreasing ΔpH*_pmf_* between lumen and stroma. In addition, the content of NADPH significantly increased, while O_2_^–^ content significantly decreased under combined application of Mg and IAA compared with Al treatment alone under spray IAA and Al treatments with or without Mg addition under none IAA spray (Figures 9B,C), indicating that the interaction of Mg and IAA regulated more electron transfer to generate NADPH rather than to generate ROS, thus, protected photosystems against oxidative damage under Al stress. Based on the results of the present study and others (Mikko and Aro, 2014; Guo et al., 2016; Wang P. et al., 2016), we constructed a model for illustrating how Mg alleviated Al-induced photoinhibition of photosystems via IAA (Figure 10).

## Conclusion

Excess Al strongly inhibited photosynthesis and photosystems of alfalfa seedlings in acid condition, but application of Mg and IAA either alone or combine increased *pmf*, CEF, g_H_^+^, ATP synthase activity, and PM H^+^-ATPase activity, and decreased ΔpH*_pmf_*, which led to increase of Pn, quantum yields, and electron transfer rates of PSI and PSII. However, these positive effects induced by Mg addition did not occur after removing apical buds of seedlings, and reappeared after spraying IAA, indicating that Mg alleviation of Al-induced photosystem damage closely depended on IAA.

## Data Availability Statement

All datasets generated for this study are included in the article/Supplementary Material.

## Author Contributions

LS contributed to conceptualization, investigation, data curation, formal analysis, and writing the original draft. AL and WW contributed to investigation and data curation. PZ contributed to data curation. YA contributed to project administration, conceptualization, funding acquisition, reviewing, and editing the manuscript.

## Conflict of Interest

The authors declare that the research was conducted in the absence of any commercial or financial relationships that could be construed as a potential conflict of interest.

## Supplementary Material

FIGURE S1 Root length **(a)**, shoot **(b)**, and root **(c)** fresh weight (FW) of alfalfa seedlings with apical buds grown in 1.5 mM Ca(NO_3_)_2_ medium (pH 4.5) containing 0 μM AlCl_3_ (pH4.5), 100 μM AlCl_3_ (pH4.5+Al), 100 μM AlCl_3_, and 25 μM MgCl_2_ (pH4.5+Al+25 μM Mg), or 100 μM AlCl_3_ and 50 μM MgCl_2_ (pH4.5+Al+50 μM Mg) at the first, third, and sixth days after the initiation of treatments. Data are means ± SE of three replicates from three independent experiments. Bars with different letters indicate significant difference at *P* < 0.05 (least significant difference test).

FIGURE S2 Chlorophyll a **(a)** and chlorophyll b **(b)** contents in leaves of alfalfa seedlings with apical buds grown in 1.5 mM Ca(NO_3_)_2_ medium (pH 4.5) containing 0 μM AlCl_3_ (pH4.5), 100 μM AlCl_3_ (pH4.5+Al), 100 μM AlCl_3_ and 50 μM MgCl_2_ (pH4.5+Al+Mg), 100 μM AlCl_3_ and 6 mg L^–1^ IAA (foliar spray) (pH4.5+Al+IAA), or 100 μM AlCl_3_ and 50 μM MgCl_2_ and 6 mg L^–1^ IAA (foliar spray) (pH4.5+Al+Mg+IAA) at the third and sixth days after the initiation of treatments. Data are means ± SE of three replicates from three independent experiments. Bars with different letters indicate significant difference at *P* < 0.05 (least significant difference test).

FIGURE S3 Light intensity dependence of photosynthetic quantum yields of Y(ND) and Y(NA) in PSI and Y(NPQ) and Y(NO) in PSII in leaves of alfalfa seedlings with or without apical buds. Five treatments in the seedlings with apical buds are as Supplementary Figure S2, and seedlings without apical buds are grown in 1.5 mM Ca(NO3)_2_ medium (pH 4.5) and treated with or without spraying IAA (pH4.5-IAA, pH4.5+IAA), 100 μM AlCl_3_ with or without spraying IAA (pH4.5+Al-IAA, pH4.5+Al+IAA), and 100 μM AlCl_3_ and 50 μM MgCl_2_ with or without spraying IAA (pH4.5+Al+Mg-IAA, pH4.5+Al+Mg+IAA). The Y(ND) **(a)**, Y(NA) **(b)**, Y(NPQ) **(c)**, and Y(NO) **(d)** were estimated from seedlings with apical buds, and Y(ND) **(e)**, Y(NA) **(f)**, Y(NPQ) **(g)**, and Y(NO) **(h)** were estimated from seedlings without apical buds at the third day after the initiation of treatments. Bars with different letters indicate significant difference at *P* < 0.05 (least significant difference test).

FIGURE S4 Value of maximum quantum efficiency (Fv/Fm) in leaves of alfalfa seedlings with apical buds grown in 1.5 mM Ca(NO_3_)_2_ medium (pH 4.5) containing 0 μM AlCl_3_ (pH4.5), 100 μM AlCl_3_ (pH4.5+Al), 100 μM AlCl_3_ and 50 μM MgCl_2_ (pH4.5+Al+Mg), 100 μM AlCl_3_ and 6 mg L^–1^ IAA (foliar spray) (pH4.5+Al+IAA), or 100 μM AlCl_3_ and 50 μM MgCl_2_ and 6 mg L^–1^ IAA (foliar spray) (pH4.5+Al+Mg+IAA) at the third and sixth days after the initiation of treatments. Data are means ± SE of three replicates. Bars with different letters indicate significant difference at *P* < 0.05 (least significant difference test).

## Abbreviations

b_6_f: cytochrome b_6_f complex
Δ pH: trans-thylakoid pH difference or proton gradient
ETRI: photosynthetic electron flow through PSI
ETRII: photosynthetic electron flow through PSII
Fd: ferredoxin (reduced)
FNR: ferredoxin NADP^+^ reductase
Fv/Fm: maximum quantum yield of primary photochemistry
g_H_^+^: proton efflux rate
I_k_: minimum saturating irradiance
O_2_^–^: superoxide anion
P700: PSI reaction center chlorophyll dimer
P700^+^: oxidized form of primary electron donor of PSI
PC: plastocyanin
*Pmf*: proton motive force
Pn: net photosynthetic rate
PQ: plastoquinone
PSI: photosystem I
PSII: photosystem II
Y(I): the quantum yield of PSI
Y(II): the effective quantum yield of PSII
Y(NA): the quantum yield of non-photochemical energy dissipation in PSI owing to a shortage of electron acceptors
Y(ND): the quantum yield of non-photochemical energy dissipation in PSI owing to a shortage of electron donors
Y(NO): the quantum yield of non-regulated energy dissipation in PSII
Y(NPQ): the quantum yield of regulated energy dissipation in PSII.
