# Supplementary figures and images for: Auxin Is Involved in Magnesium-Mediated Photoprotection in Photosystems of Alfalfa Seedlings Under Aluminum Stress
Source: Front Plant Sci. 2020 Jun 3;11:746. doi: 10.3389/fpls.2020.00746 (PMC7286060; doi:10.3389/fpls.2020.00746)

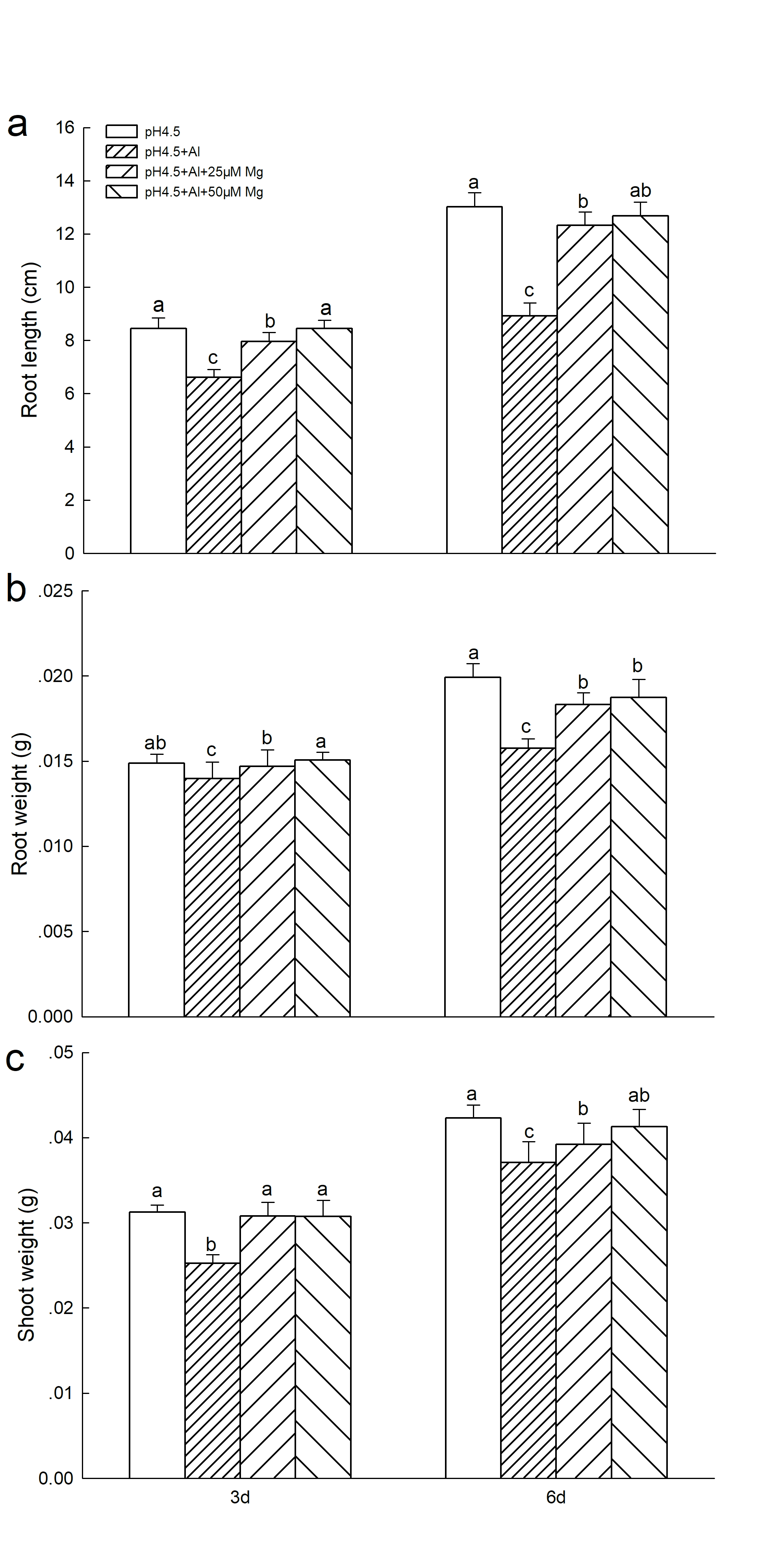

Supplement: FIGURE S1 — Root length (a), shoot (b), and root (c) fresh weight (FW) of alfalfa seedlings with apical buds grown in 1.5 mM Ca(NO3)2 medium (pH 4.5) containing 0 μM AlCl3 (pH4.5), 100 μM AlCl3 (pH4.5+Al), 100 μM AlCl3, and 25 μM MgCl2 (pH4.5+Al+25 μM Mg), or 100 μM AlCl3 and 50 μM MgCl2 (pH4.5+Al+50 μM Mg) at the first, third, and sixth days after the initiation of treatments. Data are means ± SE of three replicates from three independent experiments. Bars with different letters indicate significant difference at P < 0.05 (least significant difference test). [file Image_1.TIF]

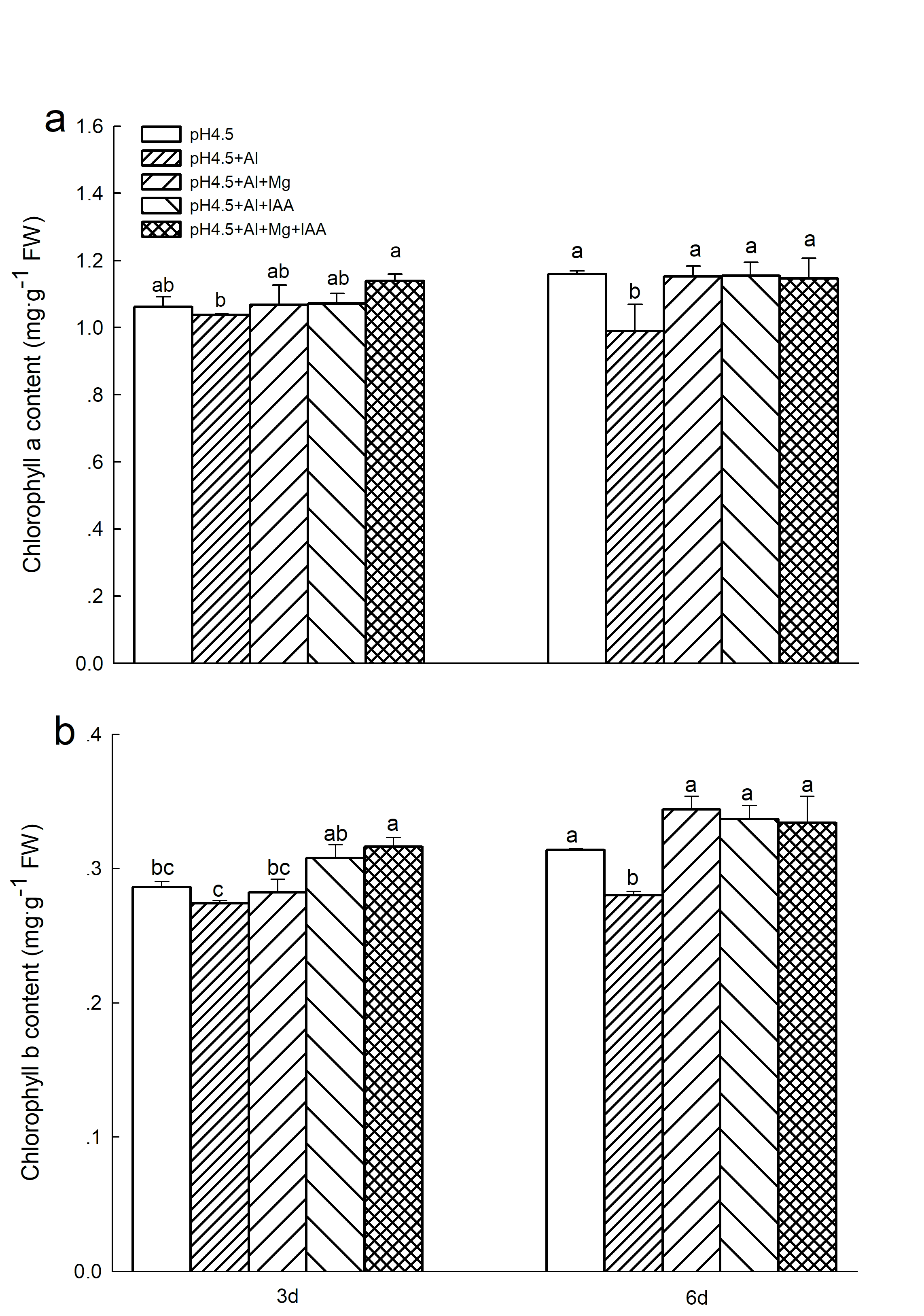

Supplement: FIGURE S2 — Chlorophyll a (a) and chlorophyll b (b) contents in leaves of alfalfa seedlings with apical buds grown in 1.5 mM Ca(NO3)2 medium (pH 4.5) containing 0 μM AlCl3 (pH4.5), 100 μM AlCl3 (pH4.5+Al), 100 μM AlCl3 and 50 μM MgCl2 (pH4.5+Al+Mg), 100 μM AlCl3 and 6 mg L–1 IAA (foliar spray) (pH4.5+Al+IAA), or 100 μM AlCl3 and 50 μM MgCl2 and 6 mg L–1 IAA (foliar spray) (pH4.5+Al+Mg+IAA) at the third and sixth days after the initiation of treatments. Data are means ± SE of three replicates from three independent experiments. Bars with different letters indicate significant difference at P < 0.05 (least significant difference test). [file Image_2.TIF]

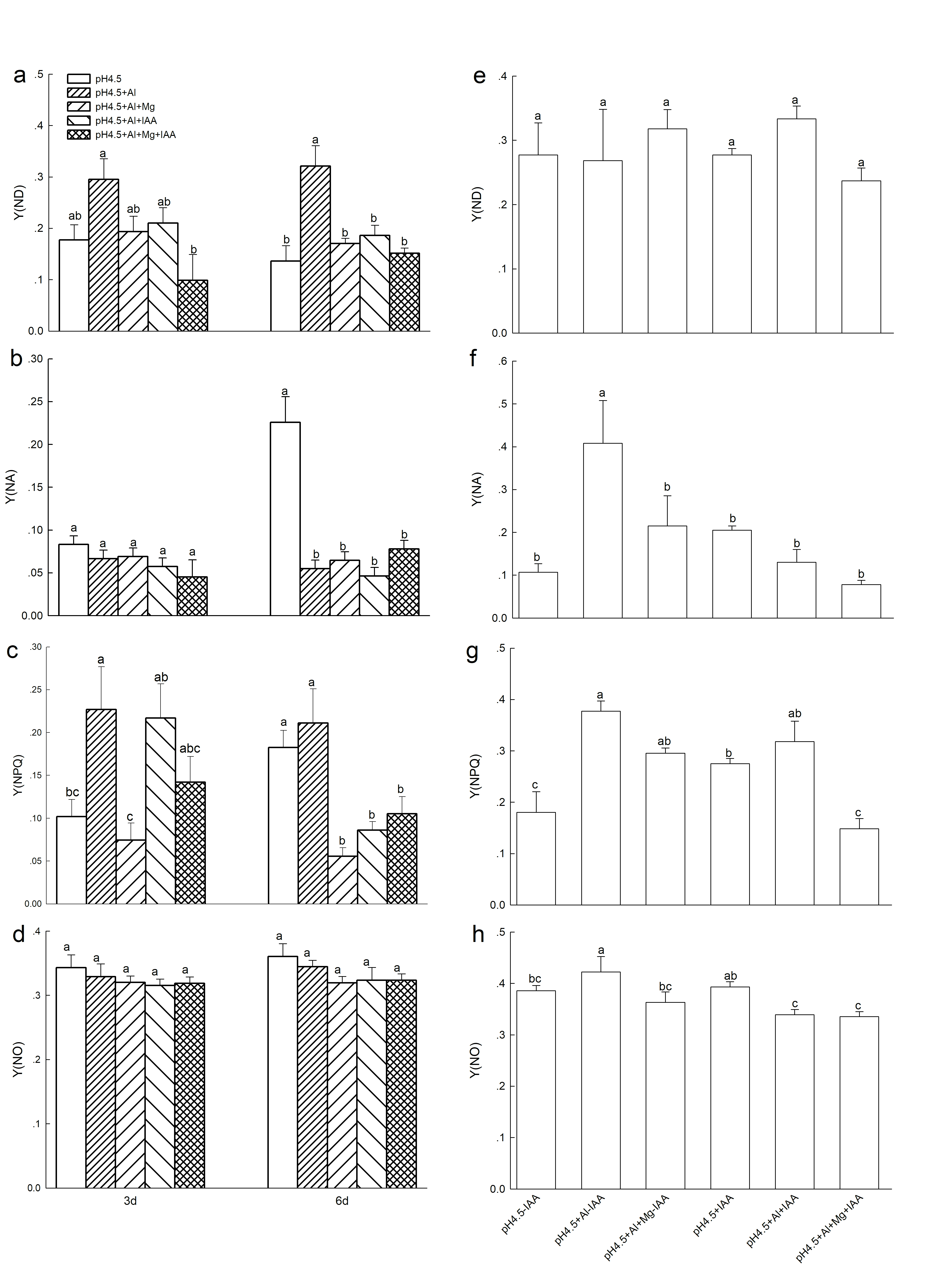

Supplement: FIGURE S3 — Light intensity dependence of photosynthetic quantum yields of Y(ND) and Y(NA) in PSI and Y(NPQ) and Y(NO) in PSII in leaves of alfalfa seedlings with or without apical buds. Five treatments in the seedlings with apical buds are as Supplementary Figure S2, and seedlings without apical buds are grown in 1.5 mM Ca(NO3)2 medium (pH 4.5) and treated with or without spraying IAA (pH4.5-IAA, pH4.5+IAA), 100 μM AlCl3 with or without spraying IAA (pH4.5+Al-IAA, pH4.5+Al+IAA), and 100 μM AlCl3 and 50 μM MgCl2 with or without spraying IAA (pH4.5+Al+Mg-IAA, pH4.5+Al+Mg+IAA). The Y(ND) (a), Y(NA) (b), Y(NPQ) (c), and Y(NO) (d) were estimated from seedlings with apical buds, and Y(ND) (e), Y(NA) (f), Y(NPQ) (g), and Y(NO) (h) were estimated from seedlings without apical buds at the third day after the initiation of treatments. Bars with different letters indicate significant difference at P < 0.05 (least significant difference test). [file Image_3.TIF]

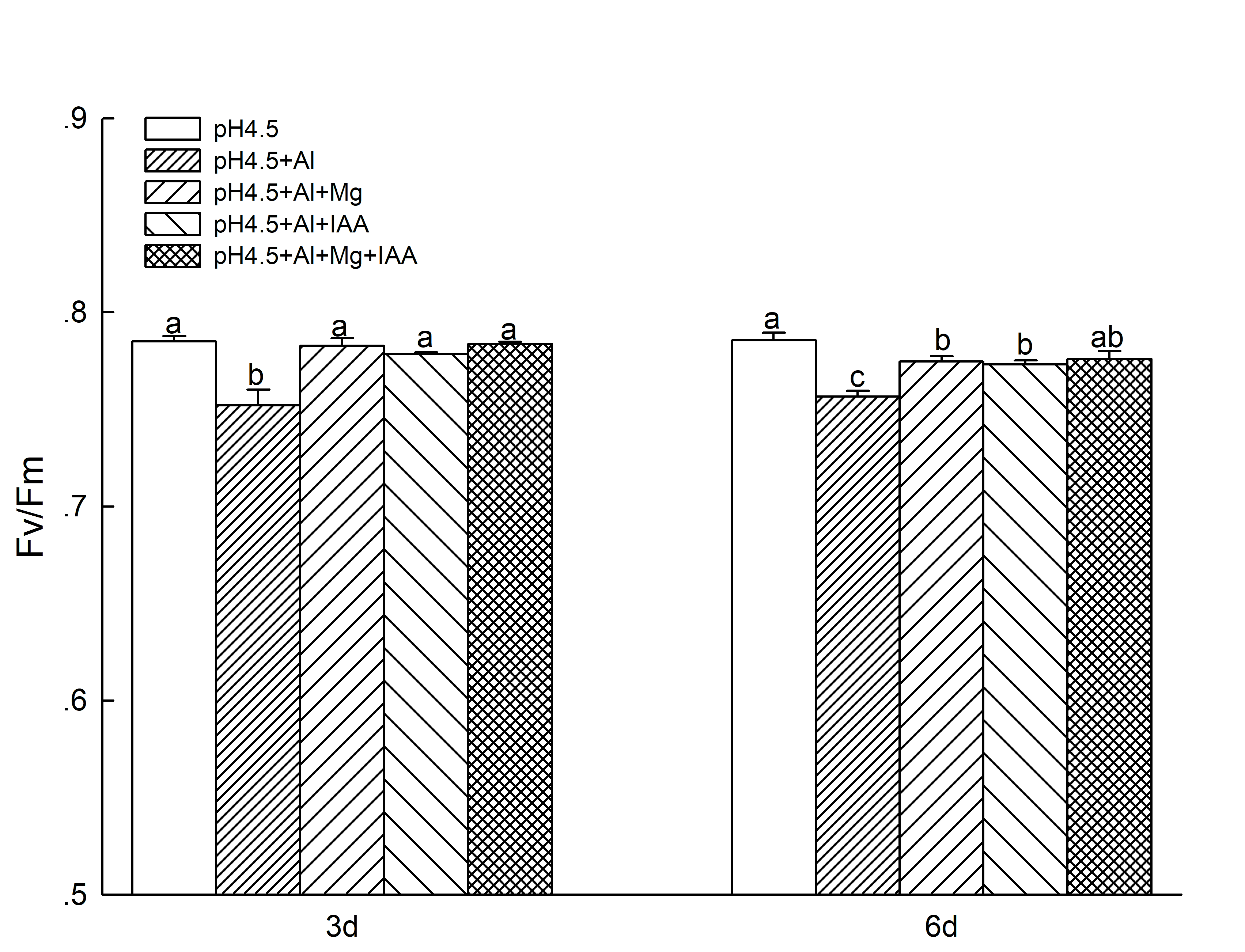

Supplement: FIGURE S4 — Value of maximum quantum efficiency (Fv/Fm) in leaves of alfalfa seedlings with apical buds grown in 1.5 mM Ca(NO3)2 medium (pH 4.5) containing 0 μM AlCl3 (pH4.5), 100 μM AlCl3 (pH4.5+Al), 100 μM AlCl3 and 50 μM MgCl2 (pH4.5+Al+Mg), 100 μM AlCl3 and 6 mg L–1 IAA (foliar spray) (pH4.5+Al+IAA), or 100 μM AlCl3 and 50 μM MgCl2 and 6 mg L–1 IAA (foliar spray) (pH4.5+Al+Mg+IAA) at the third and sixth days after the initiation of treatments. Data are means ± SE of three replicates. Bars with different letters indicate significant difference at P < 0.05 (least significant difference test). [file Image_4.TIF]
